# An Affordable Microsphere-Based Device for Visual Assessment of Water Quality

**DOI:** 10.3390/bios7030031

**Published:** 2017-08-05

**Authors:** Azra Rajwani, Brendon Restall, Nathan J. Muller, Scott Roebuck, Stephanie M. Willerth

**Affiliations:** 1 Biomedical Engineering program, University of Victoria, Victoria, BC V8W 2Y2, Canada; azra.rajwani@gmail.com (A.R.); brestall@uvic.ca (B.R.); 2 Department of Mechanical Engineering, University of Victoria, Victoria, BC V8W 2Y2, Canada; mullernathan@gmail.com; 3 Division of Medical Sciences, University of Victoria, Victoria, BC V8W 2Y2, Canada; sroebuck@uvic.ca; 4 Centre for Biomedical Research, University of Victoria, Victoria, BC V8W 2Y2, Canada

**Keywords:** water quality, global health, colorimetric assay, drug delivery, microspheres, nanoparticles

## Abstract

This work developed a prototype of an affordable, long-term water quality detection device that provides a visual readout upon detecting bacterial contamination. This device prototype consists of: (1) enzyme-releasing microspheres that lyse bacteria present in a sample, (2) microspheres that release probes that bind the DNA of the lysed bacteria, and (3) a detector region consisting of gold nanoparticles. The probes bind bacterial DNA, forming complexes. These complexes induce aggregation of the gold nanoparticles located in the detector region. The nanoparticle aggregation process causes a red to blue color change, providing a visual indicator of contamination being detected. Our group fabricated and characterized microspheres made of poly (ε-caprolactone) that released lysozyme (an enzyme that degrades bacterial cell walls) and hairpin DNA probes that bind to regions of the *Escherichia coli* genome over a 28-day time course. The released lysozyme retained its ability to lyse bacteria. We then showed that combining these components with gold nanoparticles followed by exposure to an *E. coli*-contaminated water sample (concentrations tested—10^6^ and 10^8^ cells/mL) resulted in a dramatic red to blue color change. Overall, this device represents a novel low-cost system for long term detection of bacteria in a water supply and other applications.

## 1. Introduction

The World Health Organization estimated that 842,000 deaths in lower to middle income countries are caused by inadequate water supplies, sanitation, and hygiene in 2014 [1]. Unsafe and insufficient drinking water causes approximately 502,000 of these deaths. Developing affordable and rapid methods for detecting water quality that can be easily implemented in these regions could lead to a reduction in unsafe water consumption [2]. Additionally, these systems could also be applied in isolated regions of developed countries to quickly determine whether bacteria have contaminated a water source [3]. Such water quality testing plays an important role in preventing disease outbreaks caused by contaminated infrastructure [4]. The currently accepted standard for detecting water supply contamination requires the shipment of a water sample to a laboratory where the pathogens are first cultured and then identified using standard molecular biology techniques. Ramírez-Castillo and colleagues recently reviewed these techniques [5]. These methods detect the presence of pathogen DNA or proteins in a sample, indicating that a water supply is contaminated.

Polymerase chain reaction (PCR) serves as the most commonly used technique for detecting the bacterial DNA associated with contamination [6,7]. This technique uses probes that bind to a specific region present in the pathogen genome and then polymerase enzymatically amplifies this specific region of DNA. Gel electrophoresis then confirms the presence of the pathogen specific DNA. Quantitative PCR (qPCR), a variant of this technique, uses fluorescent dyes to determine the amount of DNA present in a sample and thus can measure the levels of bacterial contamination in a sample [8]. This concept of using DNA probes to detect pathogens can be scaled up to enable the high-throughput detection process. For example, microarrays consisting of several DNA probes can identify if one or multiple pathogens are present in a water sample [9]. The detection of pathogen associated proteins serves as an alternative to these DNA based detection methods. These methods often cost more due to the use of pathogen-specific antibodies for detecting the protein epitopes [10]. Optical methods, such as UV-VIS spectroscopy and Raman spectroscopy, can also identify the presence of contaminating pathogens in a water supply [11]. Both DNA- and protein-based methods for detecting water contamination are endpoint testing methods. Accordingly, they must be repeated after resampling a water supply when monitoring for pathogen contamination over time.

Constantly monitoring a water source for contamination serves as an attractive feature for a water quality detection system. Nanotechnology offers several technologies that can be applied to monitoring water quality [11,12,13]. For example, microspheres, particles that range on the microscale (between 1 × 10^−6^ and 1000 × 10^−6^ meters), can be formed using a variety of methods, such as spray drying, solvent evaporation, single- and double-emulsion, and hot-melt microencapsulation [14]. Molecules with diverse properties have been successfully encapsulated into microspheres using a variety of different methods as reviewed in this article [14]. Controlled drug delivery provides a way to expose pathogens to the molecules necessary for detecting their DNA and proteins. Using polymeric microspheres that release DNA probes and bacteria lysing enzymes slowly over time can enable constant monitoring of a water supply for bacterial contamination. We chose to fabricate microspheres from poly (ε-caprolactone) (PCL) to deliver the different components of our novel water quality device. PCL is an inexpensive, long lasting polymer, making it an ideal choice for long-term water quality applications [15,16,17,18,19]. Its affordability also enables cost efficient production of this device for scale up manufacturing of these detection systems. Additionally, our group demonstrated that PCL microspheres can deliver bioactive proteins and small molecules for four weeks [20,21]. We have also observed similar long-term release of bioactive molecules from nanofiber mats made from PCL, providing further confirmation of its ability to generate controlled release [22,23,24]. This set of studies suggests that similar release profiles could be obtained for other biomolecules, including DNA probes for detecting bacterial DNA and lysozyme.

In terms of detecting pathogen-associated biomolecules, gold nanoparticles have been extensively investigated as sensors for biological applications [25,26]. Often these nanoparticles are conjugated with antibodies to enable colorimetric detection of bacteria [27,28]. Recently, a group showed that gold nanoparticles can visually detect the presence of DNA using hairpin probes targeting specific nucleotide sequences [29]. When the gold nanoparticles were present in a solution containing only the hairpin probes, the solution appeared red. However, when the target DNA was added to the mixture, the probes would bind to the DNA, forming complexes. These complexes induced aggregation of the gold nanoparticles, resulting in the solution becoming blue. The use of gold nanoparticles enables a highly specific, colorimetric readout for detecting DNA, which was incorporated into our device design.

**Figure 1:**
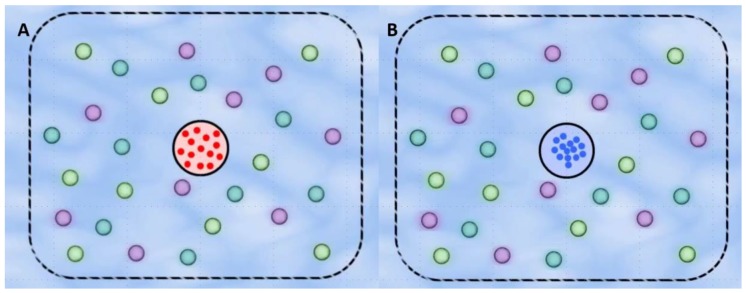
Schematic of our water quality detection prototype in the absence and presence of *E. coli* bacteria. The device contains three types of microspheres. The purple microspheres contain lysozyme, which lyses the cell walls of bacteria, releasing their DNA. The light green and blue microspheres contain the H1 and H2 DNA releasing probes, respectively. These probes bind to a specific region of the *E. coli* genome, which forms a DNA complex. (**A**) The detector region in the middle consists of gold nanoparticles which, in the absence of DNA complexes, appear red. (**B**) The presence of these DNA complexes causes the detector region shown in the middle of the device to change from red to blue as the nanoparticles aggregate.

This project develops a novel affordable, long-term water quality detection device prototype by combining biomolecule-releasing microspheres and gold nanoparticles. This device consists of enzyme-releasing microspheres that lyse bacteria present in a water sample, microspheres that release DNA probes to detect contaminating bacteria, and a detector region consisting of gold nanoparticles, as shown in Figure 1. In our device, once the bacteria are lysed and their DNA is released, they can form complexes with the probes. These complexes induce the aggregation of the gold nanoparticles through electrostatic interactions, causing a red to blue color change in the detector region observable to the naked eye. To validate our device, we fabricated microspheres containing bioactive molecules (lysozyme, an enzyme that breaks down bacterial cell walls, and two hairpin DNA probes that bind to specific regions of the *Escherichia coli* genome) using a double-emulsion method. We successfully fabricated and characterized the properties of microspheres containing these biomolecules. We then performed a study to determine the release rate for these biomolecules from the microspheres over 28 days into tap water. We next produced and characterized gold nanoparticles to serve as the detection mechanism. Finally, we combined all the elements of our system together to show that the released enzyme and probe, in combination with the nanoparticles, would change color from red to blue in the presence of water contaminated with *E. coli*, but not in the absence of bacteria. Overall, this work serves as a novel biosensor prototype for water quality detection of bacteria that could be implemented in rural areas of countries requiring continuous, affordable monitoring of water.

## 2. Materials and Methods

### 2.1. Fabrication of Microspheres Using a Double-Emulsion Process

Microspheres were fabricated using a double emulsion process, as both the hairpin DNA probes and lysozyme are hydrophilic molecules. The fabrication process for producing these double-emulsion microspheres was adapted from published methods [21,30,31]. This process required the use of two water phases (internal and external) and an oil phase. The internal/external water phases and oil phase were prepared immediately before fabrication of the double emulsion microspheres. For both sets of microspheres (DNA probes and lysozyme), the oil phase consisted of 0.54 g of PCL (Mn = 45,000, Sigma, Saint Louis, MO, USA) dissolved into 7.2 mL of dichloromethane (DCM, VWR International, Radnor, PA, USA) and 1.8 mL of methanol (MeOH, VWR International) to produce a 6% *w*/*v* solution. The external water phase consisted of a 0.25 M sodium chloride solution (NaCl, Sigma) containing 2% PVA in a total volume of 300 mL. For the lysozyme releasing microspheres, the internal water phase consisted of diluting 0.5 mg/mL of lysozyme solution (Sigma) into the 2% PVA solution to produce a final enzyme concentration of 0.25 mg/mL in 1 mL of 1% PVA.

For the microspheres containing the hairpin DNA probes, the internal water phase was prepared using 20 μM of the hairpin DNA probes (H1 sequence: CATGCCCTTCTCCCTTTGTACAAAGTTACAAAGGGAGAAG, H2 sequence: TACAAAGGGAGAAGGGCATGCTTCTCCCTTTGTAACTTTG, Integrated DNA Technologies). The hairpin nucleotide sequences were taken from [26] and the *E. coli* specific sequences were obtained from its reference genome. The DNA probe solutions were mixed at an equal ratio into a 2% PVA solution to produce a 10 μM solution in 1% PVA. The resulting solution was then well mixed before continuing with the microsphere fabrication process.

The external water phase was heated to 35 °C while being stirred at 400 rpm. The internal water phase was then emulsified into the oil phase by pipetting the internal water phase directly onto the oil phase followed by sonication for 30 s using a Model 100 sonicator (Fisher Scientific, Pittsburgh, PA, USA). The mixture was then vortexed for 15 s and sonicated for another 30 s. The emulsion was subsequently vortexed for an additional 15 s, then added drop-wise into the external water phase. The mixture was continually stirred and kept at 35 °C for 8 h to allow for the evaporation of the DCM and formation of the microspheres. After formation, the microspheres settled in solution and the remaining liquid aspirated. The microspheres were then washed with phosphate-buffered saline (PBS), centrifuged, and the PBS wash removed. They were then allowed to dry overnight before performing further characterization, as detailed in the following sections.

### 2.2. Morphological Analysis of Microspheres

Morphological analysis of double emulsion PCL microspheres was done using scanning electron microscopy (SEM) as previously published [21]. Lyophilized microsphere samples were distributed on loading stubs and coated with carbon using a Cressington 208 high-vacuum carbon coater for 6 s at 10^−4^ mbar. Images were captured using Hitachi S-4800 FE (Tokyo, Japan) scanning electron microscope using an accelerated voltage of 1.0 kV with working distances of 8.0 mm and 11.5 mm. The size of the microspheres was determined using Quartz-PCI Image Management Systems software Version 9 as performed previously [20,21].

### 2.3. Determination of Encapsulation Efficiency for the Microspheres Encapsulating Lysozyme and DNA Probes

The encapsulation efficiency represents the amount of molecule encapsulated into the microspheres relative to the initial amount present in the starting solutions used for fabrication. To measure the encapsulation efficiency, 20 mg of dried microspheres were dissolved into 750 μL of DCM and added to 750 μL of PBS within a 1.5 mL microcentrifuge tube to begin the extraction process. The tube was then vortexed at power 10 for 2 min and centrifuged (Centrifuge 5430, Eppendorf, Hamburg, Germany) at 14,000 rpm for 20 min. After this centrifugation, 200 μL of the water phase was aspirated off with a micropipette, and placed into another 1.5 mL centrifuge tube. The concentration of protein in the samples was measured using a Bio-Rad Bradford microassay protocol performed per the manufacturer’s instructions with the resulting absorbances read on an Infinite Pro 200 plate reader (Tecan, Salzburg, Austria). The concentration of oligonucleotide probes present in the solution was determined by reading the absorbance at 260 nm using a NanoVue Plus Spectrophotometer (General Electric, Boston, MA, USA).

### 2.4. Characterization of the Controlled Release of Biomolecules

We performed in vitro release studies to determine the release kinetics of lysozyme and the hairpin DNA probes from our PCL microspheres. These release studies were performed using tap water to mimic the conditions under which the device would be used. Forty milligrams of PCL microspheres containing the appropriate biomolecules were suspended in 1 mL of tap water in 1.5-mL microfuge tubes and incubated at room temperature (18–21 °C) with constant mixing using a Sarstedt Sarmix MR1 mixer. Samples were taken every two days over the course of 28 days. The sample collection process was done by first pelleting microspheres through centrifugation at 10,000 rpm for 5 min. Then 750 μL of the supernatant was removed from each tube and replaced with new solution. Each sample was stored at −20 °C. After 28 days, all samples were thawed at room temperature and subsequently measured using the Bio-Rad Bradford micro-assay protocol or using spectroscopy as described in Section 2.3.

### 2.5. Testing the Bioactivity of the Release Lysozyme

A seven-day release study was used to validate the bioactivity of the released lysozyme from the microspheres. This study used the same process as previously described to encapsulate the lysozyme in PCL microspheres and the washes were collected every day for seven days. Wild-type *E. coli* (ATCC 25922) was cultured in tryptic soy broth at 37 °C. The BacTiter-Glo™ Microbial Cell Viability Assay (Promega, Madison, WI, USA) was used to quantitatively assess the bioactivity of the lysozyme. Briefly, this kit quantifies the number of viable microbial cells in a culture by quantifying the amount of ATP present, which serves as an indicator of the metabolism of bacterial cells. The amount of ATP present is quantified using luminescence, which is then compared against a standard curve to determine the number of bacteria present. A decrease in luminescence after treatment with lysozyme indicates that the enzyme retained its activity as it shows bacterial lysis has occurred.

A 1:1 ratio consisting of 50 μL of the lysozyme washes obtained from the release study and 50 μL of *E. coli* at a concentration of 10^6^ cell/mL was incubated at 37 °C for 1.5 h on an orbital shaker to allow the lysozyme to break apart the bacterial cell walls. The 50 μL BacTiter kit was added to the assay and incubated at 37 °C for 5 min on an orbital shaker. The luminescence was read using the Infinite Pro 200 plate reader and compared to a standard curve of known lysozyme concentrations (Supplemental Figure S1).

### 2.6. Gold Nanoparticle Fabrication

Gold nanoparticles were fabricated as previously detailed using the method originally described by Frens [32,33,34]. Briefly, all nanoparticles were prepared in glassware cleaned using Aqua Regia. One millimole of HAuCl_4_ solution was boiled, followed by the addition of an amount of 38.8 mM sodium citrate equivalent to 10% of the original solution volume, causing the solution to change from yellow to colorless. Heating and mixing were continued for 10 min causing the solution to turn red. Heating was then discontinued and followed by 15 additional minutes of mixing to complete the reaction. The resulting particles were stored in amber bottles at 4 °C. Particles were then characterized using scanning electron microscopy, as described in Section 2.2.

### 2.7. Water Quality Testing Device Prototype Assembly and Evaluation

The final step of this project involved combining the different components of the device (lysozyme, H1 and H2 DNA hairpin probes, nanoparticles) to determine if a colorimetric change would be induced upon addition of *E. coli*. Bioactive lysozyme is added to break down the cell walls, releasing bacterial DNA. Then the H1 and H2 probes bind to the bacterial DNA, forming the necessary DNA complexes that induce aggregation of the gold nanoparticles. The particles will aggregate and turn from a red color (~400 nm wavelength) to a blue color (~700 nm wavelength), the color change being visible to the naked eye (Supplemental Figure S2). Our water quality detection prototype used the following concentrations of each component: 30 μL of both the 20 μM H1 and H2 DNA probe solution, 30 μL of 0.25 mg/mL lysozyme, and 180 μL of gold nanoparticles. In the absence of *E. coli*, the prototype remained visibly red. We then added 30 μL of an *E. coli* solution at concentrations of 10^6^ and 10^8^ cells/mL and observed the resulting color change. We also performed controls and demonstrated that no color change was observed when only *E. coli* and only tap water were added to the gold nanoparticles, as seen in Supplemental Figure S3.

### 2.8. Statistical Analysis

Data from samples in triplicate presented as mean values ± standard deviation of the mean. Basic correlation tests and t-tests were conducted for datasets collected from Nanovue and Infinite Pro 200 instruments. Statistical analysis was performed using MiniTab Software version 17.2.1.

## 3. Results and Discussion

### 3.1. Characterization of Lysozyme-Releasing Microspheres

**Figure 2:**
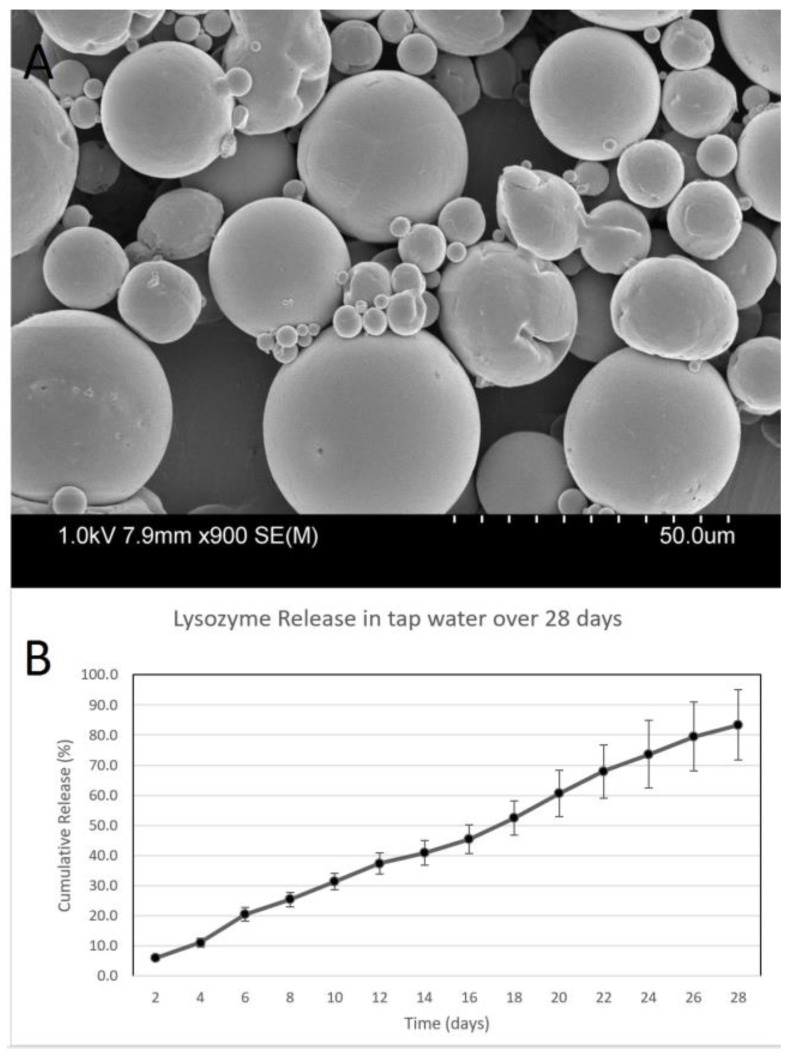
Characterization of lysozyme-releasing microspheres. (**A**) Scanning electron microscopy images of microspheres. (**B**) Graph showing the cumulative release of lysozyme over 28 days with error bars representing the cumulative standard deviation at each time interval (*n* = 3).

Figure 2A shows the morphology of microspheres encapsulating lysozyme produced using a double-emulsion method as determined using scanning electron microscopy. The shape and smooth surface morphology are consistent between the individual microspheres with some variation in size. However, some portions of PCL did not form microspheres, as shown in the image. The size of the microspheres averaged 232 ± 11 μm (Table 1). The encapsulation efficiency of lysozyme was found to be 32 ± 7%, meaning the 32% of the protein added initially was retained in the final microsphere formulation. While this number represents an acceptable encapsulation efficiency for PCL, higher efficiencies of ~80% have been observed when using poly (lactic co-glycolic acid) (PLGA) to fabricate lysozyme-releasing microspheres [35]. The amount of protein encapsulated was consistent with previous work from our group that encapsulated glial-derived neurotrophic factor (GDNF) in PCL microspheres [16]. In terms of the size distribution, it is more desirable to have larger microspheres as they enable a longer period of controlled release compared to the smaller microspheres observed using SEM. The cumulative release shown in Figure 2B indicates the successful release of lysozyme over a 28-day period in tap water. The total amount of protein released was 83.6 ± 11.6%. The overall kinetics indicate a release profile correlating to simple diffusion from the double-emulsion microspheres as the protein was released steadily with no burst release observed. These release results are consistent with the release of bovine serum albumin from PCL microspheres observed in our previous study where 88% of the total BSA was released over a comparable time course [21]. Similar release rates of proteins from PCL scaffolds has been observed by other groups [36,37]. These results are expected as this process relies on diffusion based release. The inherent material properties of PCL and the internal/surface topography of the PCL microspheres control the release rate in such delivery systems.

**Figure 3:**
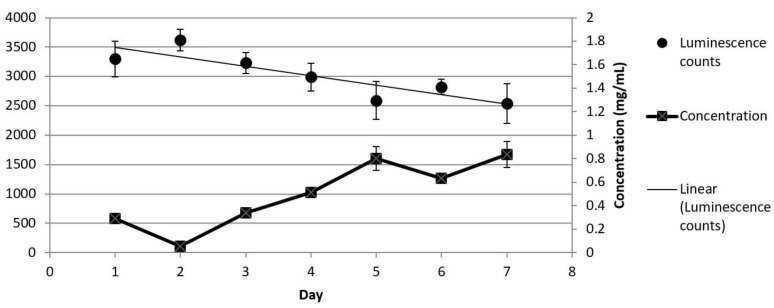
Quantification of bacteria lysis induced by lysozyme washes using the BacTiter kit. The bioactivity of the released lysozyme was assessed using the washes collected over seven days. The graph shows the relationship between the luminescence counts and lysozyme concentration for each day, which was determined from a standard curve of known lysozyme and its ability to lyse *E. coli*.

A second seven-day release study was performed to confirm that the released protein was bioactive. These collected samples containing lysozyme were used to treat known concentrations of *E. coli* with the amount of lysis measured and quantified using a known lysozyme standard curve (Supplemental Figure S1). These results are shown in Figure 3. While all washes induced a certain amount of bacterial lysis, the lysozyme released at later time points corresponded to a higher concentration of active protein compared to the earlier time points. For these studies, only the protein activity of was monitored. It is possible that the sample collected the first day had additional lysozyme present on the surface of the microspheres. This possibility would explain the decrease in enzyme activity on Day 2. As a significant amount of bacterial lysis could still be observed on Day 7, these results suggest that encapsulation of lysozyme within microspheres does preserve its enzymatic activity. These results are consistent with other work on encapsulating lysozyme into other polymers, including methods using PGLA, gelatin, and alginate [35,38,39,40].

### 3.2. Characterization of Hairpin DNA-Releasing Microspheres

**Figure 4:**
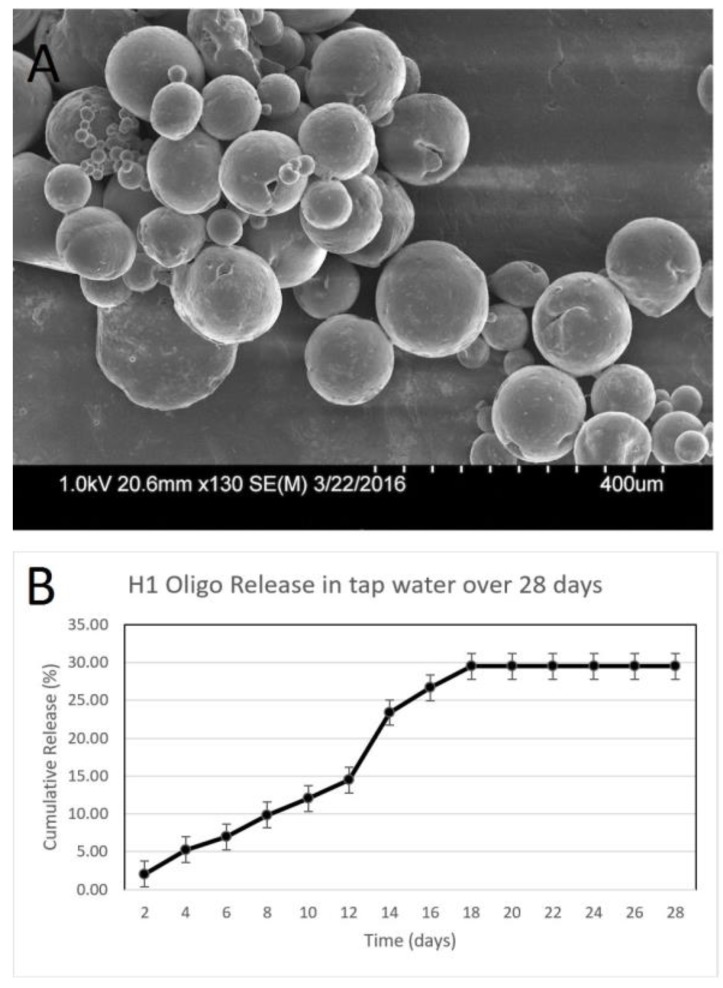
Characterization of H1 Oligo releasing microspheres. (**A**) Scanning electron microscopy images of microspheres. (**B**) Graph showing the cumulative release of the H1 DNA probe over 28 days representing the cumulative standard deviation at each time interval (*n* = 3).

**Figure 5:**
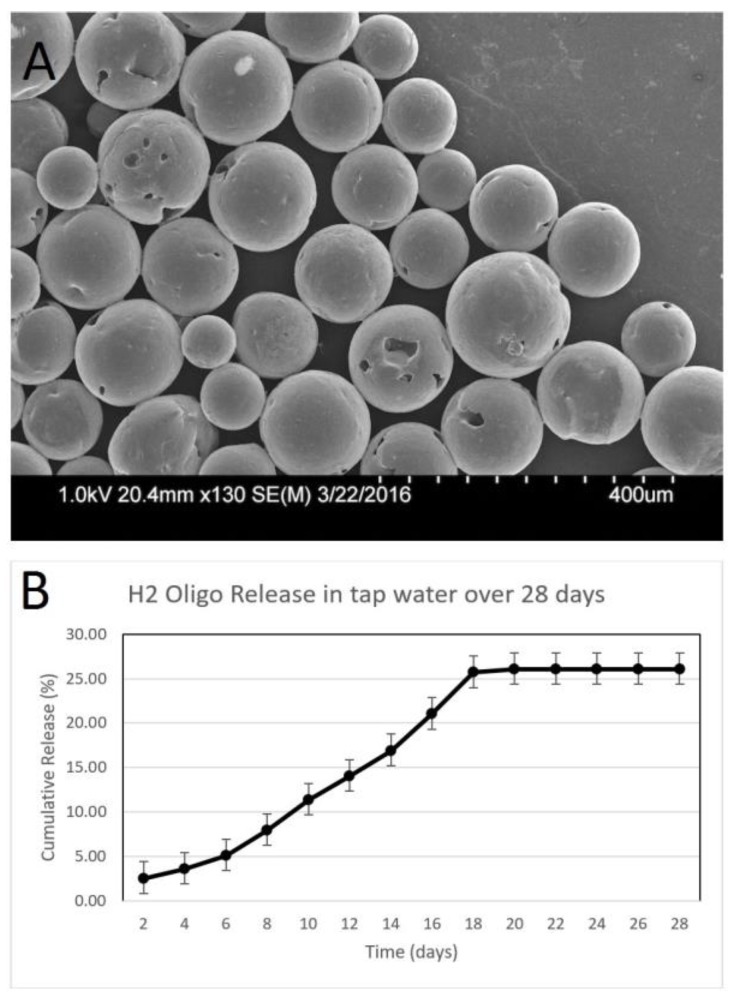
Characterization of H2 oligo-releasing microspheres. (**A**) Scanning electron microscopy images of microspheres. (**B**) Graph showing the cumulative release of the H2 DNA probe over 28 days representing the cumulative standard deviation at each time interval (*n* = 3).

Microspheres were fabricated containing the H1 and H2 hairpin DNA probes, which bind to specific regions of the *E. coli* genome, followed by characterization using scanning electron microscopy (Figure 4A and Figure 5A). These microspheres exhibited regions of surface roughness while maintaining a spherical morphology. The microspheres containing the H1 probe averaged 243 ± 17 μm in diameter while the microspheres containing the H2 probes averaged 227 ± 13 μm as listed in Table 1. The encapsulation efficiency was 35 ± 2% and 49 ± 2% for the H1 and H2 microspheres, respectively. The H1 microspheres released 30 ± 2% amount of probe over 28 days into tap water (Figure 4B) while the H2 microspheres released 26 ± 2% over the same time (Figure 5B). Unlike with lysozyme, the DNA release appears to plateau around Day 18. The different release profiles could be due to the difference in the size between these two biomolecules. The hairpin DNA molecules possess a significantly lower molecular weight in comparison with the much larger lysozyme protein. Their electrostatic properties also play a role in these altered release patterns.

Another group achieved efficient delivery of DNA from PCL microspheres by first encapsulating these molecules into gelatin nanoparticles, followed by incorporation into larger microspheres [41]. This method of fabrication yields smaller microspheres (~1–10 μm in diameter) that released the incorporated DNA over the course of hours. The use of gelatin nanoparticles also resulted in an encapsulation efficiency of ~93%, which is much higher than our reported values. Thus, it would be worthwhile to explore alternative methods and polymers for delivery of these hairpin DNA probes. Finally, sieving could be used to achieve a more uniform microsphere size distribution for both types of microspheres and remove excess PCL.

### 3.3. Gold Nanoparticle Synthesis

**Figure 6:**
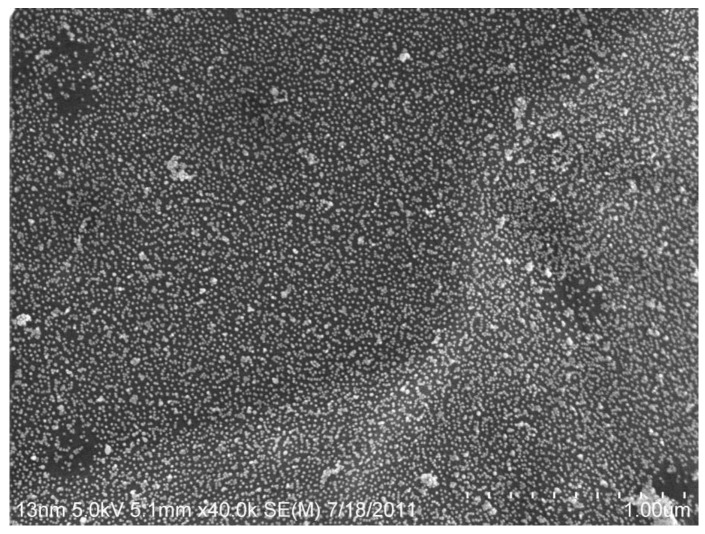
Scanning electron microscopy images of gold nanoparticles immobilized on a surface. We observed consistently-sized gold nanoparticles. The Scale bar is 1 μm.

Gold nanoparticles were synthesized as previously described [32,33]. Scanning electron microscopy was performed to confirm successful fabrication of the gold nanoparticles (Figure 6). The particles were in the nanoscale size range and exhibited typical nanoparticle morphology. We confirmed that DNA complexes consisting of the bacterial probes bound to purified *E. coli* DNA caused these particles to aggregate, inducing a visual color change from red to blue (Supplemental Figure S2). Our observations were consistent with previously-published work [29,42].

### 3.4. Prototype Validation

**Figure 7:**
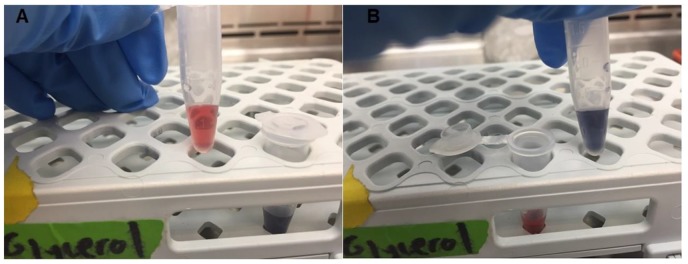
Validation of our water quality detection device prototype. (**A**) Eppendorf tube containing the DNA probes, lysozyme, and gold nanoparticles with no bacteria present. (**B**) Eppendorf tube containing the DNA probes, lysozyme, and gold nanoparticles with *E. coli* present (concentration: 10^8^ cells/mL). The change in color from (**A**) (red) to (**B**) (blue) is visually identifiable.

For the final step of this project, we combined these components to create a device prototype to verify if a colorimetric change would be induced upon addition of *E. coli* to the system. Figure 7 shows the prototype of our water quality device being tested to detect the presence of *E. coli* in a water sample. In Figure 7A,B, the necessary components of our water quality detection system are present (lysozyme, H1 and H2 DNA probes, and gold nanoparticles). In Figure 7B, *E. coli* was added to the system, inducing aggregation of the gold nanoparticles, turning the solution from a red color to a blue color. We also observed a similar color change when using a lower concentration of bacteria (10^6^ cells/mL, Supplemental Figure S4). As mentioned in the methods, controls consisting of only tap water and only *E. coli* did not induce this color change, suggesting that our system worked as intended. The work detailed in the paper provides a novel way to detect the presence of bacterial contamination in water while continuously monitoring water quality over time through the controlled release of biomolecules.

This work, however, represents only a crucial first step in this process, demonstrating how such a system could be feasible for water quality monitoring. One of the most important features of our prototype is that the use of microspheres makes the device modular. Thus, it would be convenient to adapt this system for detecting other types of bacteria simply by changing the sequences of the hairpin DNA probes based on the target pathogen. The use of gold nanoparticles enables simple, colorimetric visualization of contamination, avoiding the need for expensive lab equipment. The combination of controlled release and nanotechnology makes this device a novel biosensing system.

Many issues must first be addressed to further translate this work into practical application. Here we only tested its ability to detect relatively high concentrations of *E. coli* and, thus, future work could evaluate how sensitive our device is in terms of a minimum concentration threshold of detected bacteria. Another potential issue is the possibility of false positives, which may occur in water supplies that have other solutes present that could interfere with the nanoparticle chemistry used in this paper. However, we showed that tap water alone, nor does *E. coli* alone, trigger a false positive as seen in Supplemental Figure S3. The release studies suggest we can obtain the release of the probes and lysozyme consistently over an 18-day period, though optimizing the fabrication process for the microspheres that release the DNA probes could potentially increase this time course. These microspheres could be fabricated from another polymer or compatible material that would allow for a more consistent extended release pattern to increase the utility of the device. Additionally, there is room to improve by increasing the encapsulation efficiency of these microspheres, which might contribute to a longer release time course which, in turn, would extend the lifespan of the device.

## 4. Conclusions

We developed a novel prototype of an affordable, long-term water quality biosensing device that uses microspheres to deliver both lysozyme and DNA probes in combination with gold nanoparticles for detecting the presence of *E. coli*. in a water sample. The lysozyme breaks down the cell walls of the *E. coli*, releasing its DNA. The DNA probes then bind to the *E. coli* DNA and these complexes cause the gold nanoparticles to aggregate. When the gold nanoparticles aggregate, the solution undergoes a visual color change from red to blue, providing a visual indicator of contamination. Here we demonstrate that such a system can detect an *E. coli* concentration of 10^8^ cells/mL. Overall, the work performed in this study provides a novel and modular method for detection of bacterial pathogens present in a water supply. It also serves as an alternative to traditional laboratory based methods for pathogen detection that require expensive equipment and significant labor.

## Figures and Tables

**Table 1:** Properties of biomolecule-releasing microspheres.

Molecule Encapsulated	Average Diameter	Encapsulation Efficiency	Percentage Released after 28 Days
Lysozyme	232 ± 11 μm (*n* = 46)	32 ± 7% (*n* = 3)	83 ± 11% (*n* = 3)
H1 DNA probe	243 ± 17 μm (*n* = 24)	35 ± 2% (*n* = 3)	30 ± 2% (*n* = 3)
H2 DNA probe	227 ± 13 μm (*n* = 30)	49 ± 2% (*n* = 3)	26 ± 2% (*n* = 3)

## Supplementary Materials

The following are available online at www.mdpi.com/2079-6374/7/3/31/s1; **Figure S1.** Standard curve evaluating the ability of different lysozyme concentrations ranging from 1 mg/mL to 0.001 mg/mL to lyse bacteria using the BacTiter kit. The amount of viable bacteria decreases as the amount of lysozyme increases as expected. The R^2^ value for the regression was 0.9693. **Figure S2.** Gold nanoparticles change color in the presence of decreasing concentrations of DNA complexes. The image showing the spectrum of gold nanoparticles in the presence of decreasing concentration of DNA complexes. The dark blue tube on the far left has the highest concentration of bacterial DNA combined with the H1 and H2 oligos (concentration: 10 μM), which is then diluted 10-fold in each adjacent tube while the tube containing no complexes appears red on the far right. **Figure S3.** Controls for the device prototype. The first tube on the left contains a mixture of tap water and gold nanoparticles and it appears red. The middle tube contains gold nanoparticles with no additional components present, which serve as a negative control. The tube on the right contains *E. coli* along with the gold nanoparticles and it appears red. **Figure S4.** Validation of our water quality detection device prototype. The Eppendorf tube containing the DNA probes, lysozyme, and gold nanoparticles with no bacteria present is on the right. The Eppendorf tube containing the DNA probes, lysozyme, and gold nanoparticles with *E. coli* present (concentration: 10^6^ cells/mL) is on the left. The change in color from red to blue is visually identifiable.
