# Supplementary material for: An Affordable Microsphere-Based Device for Visual Assessment of Water Quality
Source: Biosensors (Basel). 2017 Aug 5;7(3):31. doi: 10.3390/bios7030031 (PMC5618037; doi:10.3390/bios7030031)
Supplement: Supplementary file 1 [file biosensors-07-00031-s001.pdf]

Supplementary Materials: An Affordable Microsphere-Based Device for Visual Assessment of Water Quality

Azra Rajwani, Brendon Restall, Nathan J. Muller, Scott Roebuck and Stephanie M. Willerth

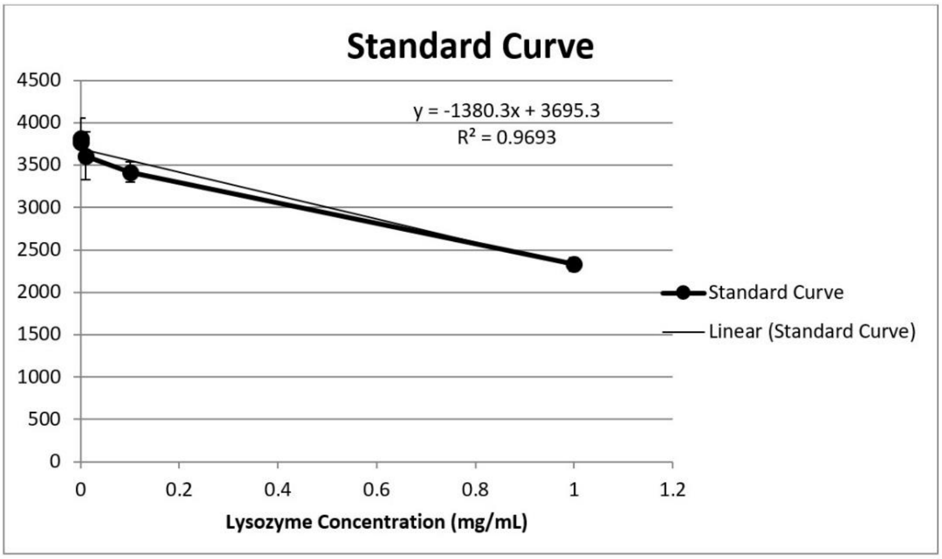

Figure S1. Standard curve evaluating the ability of different lysozyme concentrations ranging from 1 mg/mL to 0.001 mg/mL to lyse bacteria using the BacTiter kit. The amount of viable bacteria decreases as the amount of lysozyme increases as expected. The R^2 value for the regression was 0.9693.

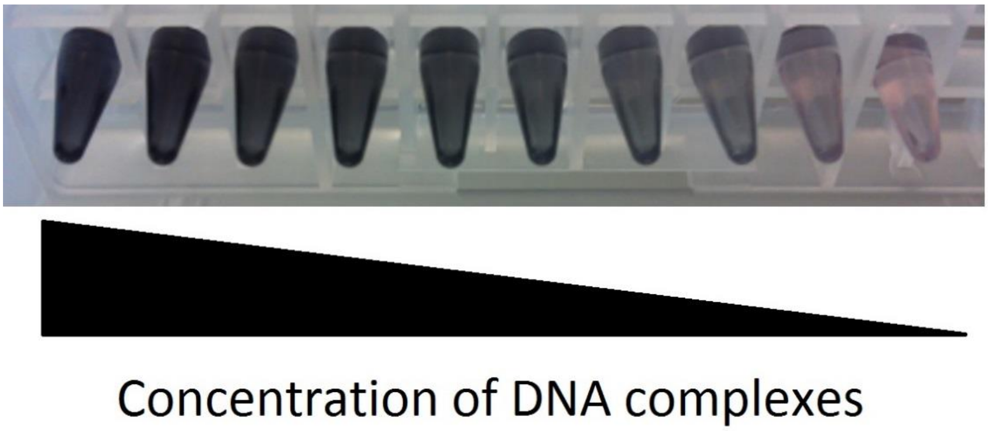

Figure S2. Gold nanoparticles change color in the presence of decreasing concentrations of DNA complexes. The image showing the spectrum of gold nanoparticles in the presence of decreasing concentration of DNA complexes. The dark blue tube on the far left has the highest concentration of bacterial DNA combined with the H1 and H2 oligos (concentration: 10 µM), which is then diluted 10-fold in each adjacent tube while the tube containing no complexes appears red on the far right.

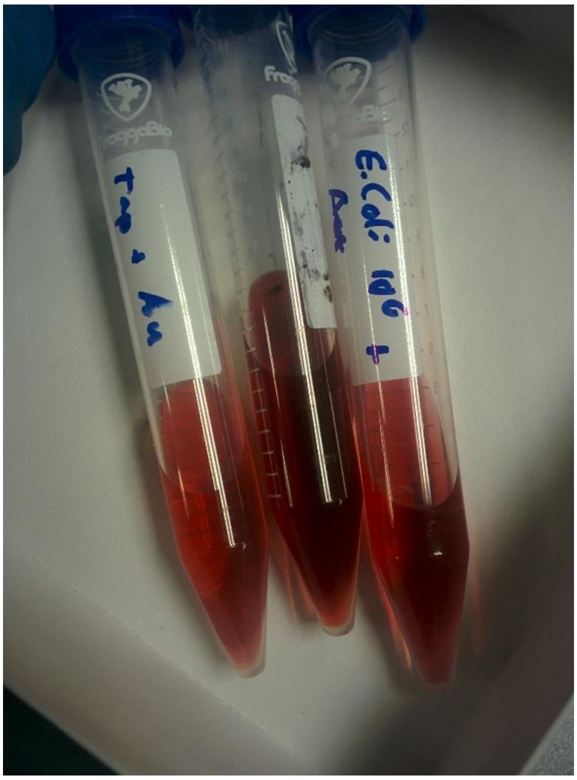

Figure S3. Controls for the device prototype. The first tube on the left contains a mixture of tap water and gold nanoparticles and it appears red. The middle tube contains gold nanoparticles with no additional components present, which serve as a negative control. The tube on the right contains *E. coli* along with the gold nanoparticles and it appears red.

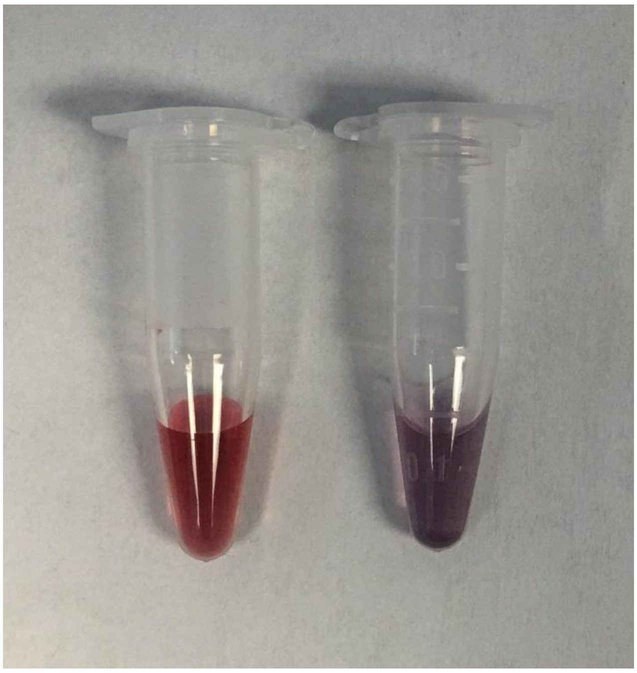

Figure S4. Validation of our water quality detection device prototype. The Eppendorf tube containing the DNA probes, lysozyme, and gold nanoparticles with no bacteria present is on the right. The Eppendorf tube containing the DNA probes, lysozyme, and gold nanoparticles with *E. coli* present (concentration: 10^6 cells/mL) is on the left. The change in color from red to blue is visually identifiable.
